# Constructing Stable Polyvinyl Alcohol/Gelatin/Cellulose Nanocrystals Composite Electrospun Membrane with Excellent Filtration Efficiency for PM2.5

**DOI:** 10.3390/polym16121656

**Published:** 2024-06-11

**Authors:** Yang He, Haijun Liu, Weijun Ying

**Affiliations:** 1 Jiyang College of Zhejiang Agriculture and Forestry University, Shaoxing 311800, China; 20080031@zafu.edu.cn; 2 Huzhou College, Huzhou 313002, China; haijunliu@126.com

**Keywords:** polyvinyl alcohol, gelatin, electrospun, air filtration membrane, filtration efficiency

## Abstract

Considering the high demand for air quality, the development of biomass-based air filtration membranes with high air filtration efficiency and good stability is an urgent task. In this work, polyvinyl alcohol (PVA), gelatin (GA), and cellulose nanocrystals (CNC) were mixed and prepared into a membrane through an electrospinning method for air filtration. After a hydrophobic modification, the modified PVA/GA/CNC composite membrane showed excellent filtration efficiency for PM2.5 (97.65%) through the internal three-dimensional structure barrier and the electrostatic capture effect of the CNC with a negative charge, as well as a low-pressure drop (only 50 Pa). In addition, the modified PVA/GA/CNC composite membrane had good mechanical properties (maximum tensile fracture rate of 78.3%) and high stability (air filtration efficiency of above 90% after five wash-filter cycles and a high-temperature treatment at 200 °C). It is worth noting that the whole preparation process is completed without organic solvents, putting forward a new strategy for the construction of green air filtration membranes.

## 1. Introduction

With the rapid development of global industry, air pollution has become a particularly serious problem [1,2,3]. Air pollution includes toxic and harmful gas emissions [4], particulate matter pollution [5], etc. Of all, particulate matter (PM), especially particulate matter with a diameter of less than 2.5 μm (PM2.5), is known to be the most serious, which poses a great threat to human health and social order [6,7,8,9,10]. It is reported that a large number of people and animals are sick or die because of air pollution every year, which is particularly obvious in some rapidly growing industrial areas [11,12]. At the same time, this phenomenon is associated not only with industrial production, transportation, and waste processing but also with daily family activities, such as cooking, heating equipment, and fireworks [13,14]. The membrane for air filtration with effective PM2.5 purification has attracted much attention, which is of great significance for environmental protection and sustainable industrial development [15,16,17].

Excellent air filters require high filtration efficiency while maintaining low-pressure drop, which is conducive to reducing energy consumption and high-value application [18,19]. Currently, there is a wide range of air filtration materials, including non-woven fabrics, aerogels, membranes, etc. Wang et al. used a dip-coating method combining non-solvent-induced phase separation and surface deposition to prepare thin, layered, porous environmental protection non-woven fabrics with a filtration efficiency of 96.8% for PM2.5 and a pressure drop of 143.9 Pa [20]. Lyu et al. dissolved hemp fiber in a precooled sodium hydroxide/urea system to form a composite aerogel with a PM2.5 filtration efficiency of 94% and low-pressure drop [21]. As is known, aerogels usually are of poor mechanical strength and have a limitation on their application range. Wang et al. used a direct foaming method combined with a belt casting method to prepare mullite-bonded sic ceramic porous foam membrane with a three-dimensional interconnected pore network, only 1 mm in thickness, a filtration efficiency of 90.1% for PM2.5, and a pressure drop of 27 Pa [22]. However, the belt casting method is not only cumbersome but the filtration efficiency still needs improvement.

Electrospun membranes are different from air filtration materials such as non-woven fabrics and cast membranes [23,24,25]. They have a regular controllable diameter of nano-fibrils and are ideal materials for efficient PM2.5 purification systems. In recent years, due to the adhesiveness, flexibility, and extensibility of PVA, there has been widespread interest in PVA electrospun membranes. The stability of pure PVA electrospun membrane is usually poor, but it can be improved by introducing other polymers to PVA. Li et al. used a method combining polypropylene (PP) melt-blown with PVA/zeolite imidazole framework-8 (ZIF-8) electrospinning to construct a PP/PVA/ZIF-8 composite membrane with a layered fiber structure, showing high PM2.5 filtration efficiency (96.5%) [26]. The poor mechanical properties of PVA-based electrospinning membranes always affected their application. Cui et al. prepared PVA-LS composite membranes by electrospinning and thermal crosslinking of PVA with sodium lignosulfonate (LS), demonstrating significant improvement of air filtration performance and mechanical properties, and the strong air filtration performance can still be maintained after 10 filtration cycles [27]. However, the glass transition temperature of PVA is about 75~85 °C. This means that within this temperature range, PVA begins to transition from a hard and brittle glassy state to a soft, rubbery state, which greatly limits its application in high-temperature environments [28]. Therefore, the development of a PVA-based electrospinning air filtration system with good thermal stability and high PM2.5 filtration capability is required [29,30,31].

This study reports a PVA-based electrospun membrane system with a high filtration capacity for PM2.5 and excellent heat resistance. In the system, PVA was used as the main spinning substrate, which was mixed with gelatin (GA) to enhance the thermal and with cellulose nanocrystals (CNC) to improve the filtration performance of the PVA/GA/CNC composite membrane. Then, this composite membrane was hydrophobized with methyltrimetoxysilane (MTMS) using the chemical vapor deposition method. Due to the compact three-dimensional network structure and the electrostatic attraction of CNC to PM2.5, the modified PVA/GA/CNC composite membrane shows a high PM2.5 filtration efficiency (97.65%) and low-pressure drop (50 Pa), maintaining the filtration performance of more than 90% after five wash-filtration cycles and high-temperature treatment at 200 °C. In addition, the whole process is carried out without an organic solvent, which has a high potential for application while protecting the environment.

## 2. Experimental Section

### 2.1. Materials

Polyvinyl alcohol (PVA, 17–99 L, Mw: 74,800–79,200, the alcoholysis degree is 98–99 mol/mol) was obtained from Yuan Ye Biotechnology (Shanghai, China). Aqueous 10% solution of gelatin (GA, Mw: 261.23) and methyltrimethoxysilane (MTMS, 98%, Mw: 136.22) were purchased from Shanghai Macklin Biochemical Technology Co., Ltd. (Shanghai, China). Aqueous 5.44% dispersion of cellulose nanocrystals (CNC, Purity > 99%, particle size of 100–200 nm, Zeta potential of −36–39 mV) was purchased from Tianjin Muzhiling Biotech Co., Ltd. (Tianjin, China). All chemicals were used without further treatment.

### 2.2. Preparation of Polyvinyl Alcohol/Gelatin/Cellulose Nanocrystals Composite Membrane

A PVA solution with a concentration of 10% was prepared before electrospinning by dissolving it in deionized water. Then, certain amounts of PVA and GA solutions and CNC dispersion were mixed at 100 °C and stirred for 1 h to form a PVA/GA/CNC liquid mixture. Finally, this liquid mixture was placed in the needle tube, and electrospinning was carried out at a voltage difference of 20 kV, with the needle 15 cm away from the receiving hot plate to obtain PVA/GA/CNC composite membrane used as an air filter. The composite membranes of different proportions are labeled PVA/GAx/CNCy (PVA:GA:CNC = 1:x:y).

### 2.3. Preparation of Modified Polyvinyl Alcohol/Gelatin/Cellulose Nanocrystals Composite Membrane

Hydrophobic modification of the PVA/GA/CNC composite membrane was performed using the vapor deposition method of MTMS. Briefly, a small glass bottle containing 1 mL of MTMS was placed in a closed large glass bottle with a PVA/GA/CNC composite membrane and heated at 120 °C for 4 h. Finally, the modified PVA/GA/CNC composite membrane was removed from the large glass bottle and heated for 1 h to remove the unreacted MTMS.

### 2.4. Filtration Capacity Test

**Figure 1:**
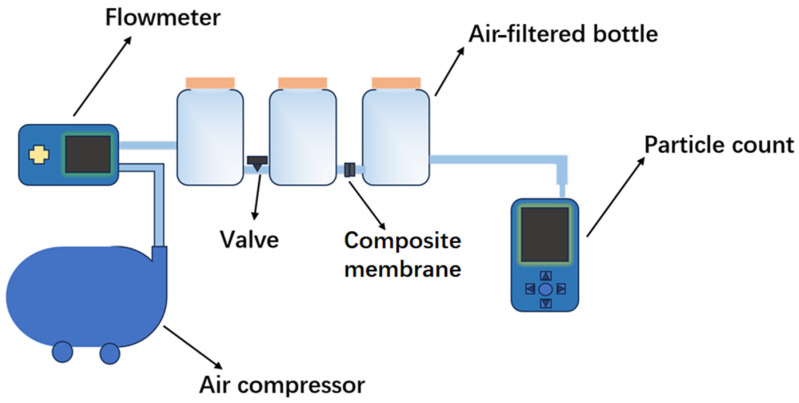
Scheme of self-built PM2.5 filtration set.

According to a previous report [32,33], the flow meter monitors the flow of air that may contain smoke generated by burning incense (Figure 1). The particle counter calculates the concentration of 2.5 µm particles remaining after filtration through the composite membrane. In this system, the PM2.5 concentration is controlled to be >500 μg/m^3^. PM2.5 filtration efficiency was calculated according to the formula E = (N_0_ − N_1_)/N_0_, where N_0_ and N_1_ were the number of particles measured without and with samples, respectively. All tests were repeated three times and averaged. The pressure drop is measured by a differential pressure gauge (German Testo 510, FTB Mfg. Co., Ltd., Taichung, Taiwan). The mass fraction formula Qf = −ln(1 − E)/ΔP was used to evaluate the filtration effect of PM2.5, where E is taken as the filtration efficiency of PM2.5, and ΔP as the pressure drop.

### 2.5. Heat Resistance Test

The electrospun membranes were dried at 25 °C, 50 °C, 100 °C, and 150 °C for 2 h, and then filtration efficiency and pressure drop were tested to determine thermal stability.

### 2.6. Mechanical Property Test

The mechanical properties of different electrospun membranes were tested by a CMT6104 universal strength testing machine. The standard sample size was fixed as 40 × 10 × 0.1 mm (length × width × thickness), and the test results of 5 samples in each group were averaged. The stretching speed was 5 mm/min, and the clamping distance was 20 mm.

### 2.7. Characterization

A scanning electron microscope (FEI, Hillsboro, OR, USA) was used to observe the micro-morphology of various electrospun membranes. Chemical and elemental analysis of various electrospun membranes was performed by Fourier transform infrared spectroscopy (Nicolet IS5, Tampa, FL, USA) with a scanning range of 4000–400 cm^−1^. A tester (Shanghai Zhongchen JC2000D, Shanghai, China) was used to test the water contact angle (WCA) and rolling angle (SA) of electrospun membranes. Each sample was tested 5 times, and the average value was taken. The thermal stability of electrospun membranes was tested using a thermogravimetric analyzer (TGA, STA 499 F5 Jupiter, Berlin, Germany) in a nitrogen (N_2_) environment. The heating rate is 10 °C/min, and the temperature is 30 °C to 800 °C.

## 3. Results and Discussion

### 3.1. Formation and Working Proposed Mechanism of Modified Polyvinyl Alcohol/Gelatin/Cellulose Nanocrystals Composite Membrane

**Figure 2:**
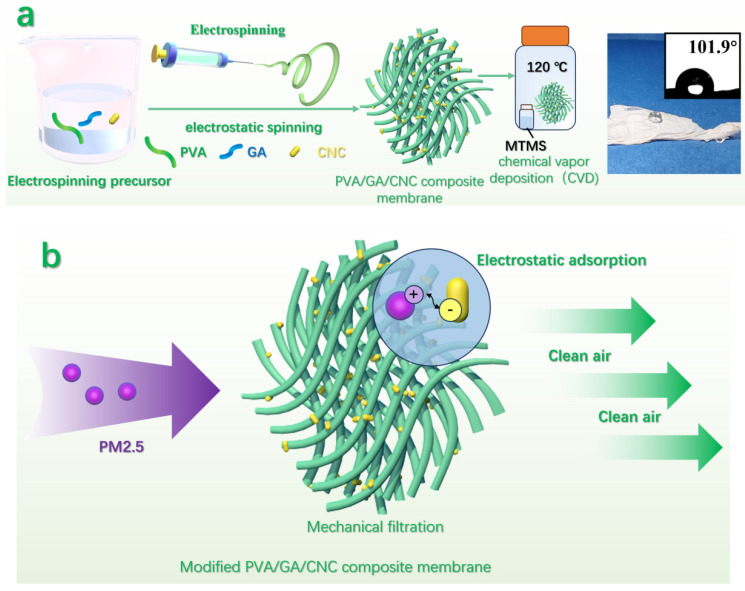
(**a**) Schematic diagram of formation and (**b**) working proposed mechanism of modified PVA/GA/CNC composite membrane.

As shown in Figure 2, the PVA/GA/CNC composite membrane was obtained by electrospinning of liquid mixtures containing PVA, GA, and CNC. Then, the composite membrane was treated with a hot gas phase of MTMS to hydrophobize it.

In the system, PVA was used as the main material for constructing composite membranes. Because GA is of high viscosity and good compatibility, its introduction can not only keep the green characteristics of a system, but also further improve the mechanical properties and thermostability. As a reinforcing material, CNC has a good dispersing due to its rich surface charge. Meanwhile, CNC is connected with the active hydroxyl on the surface of PVA and GA by hydrogen bonds, making its combination more stable. The modified PVA/GA/CNC composite membrane blocks the passage of PM2.5 through its rich three-dimensional network structure. In addition, the -HSO_3_ groups formed during the CNC production process provide a negative zeta potential of −38 mV. As a result, the negatively charged cellulose nanocrystals of the membrane attract positively charged PM2.5 particles and thereby promote the removal of these particles [31]. Due to a dual PM2.5 filtration mechanism by the porous structure of the membrane and electrostatic attraction, the modified composite membrane exhibits a high filtration efficiency of solid particles with a low-pressure drop. It is worth noting that MTMS deposited on the surface of the composite membrane in the form of long chains provides a high degree of hydrophobization.

### 3.2. Microstructure Analysis

**Figure 3:**
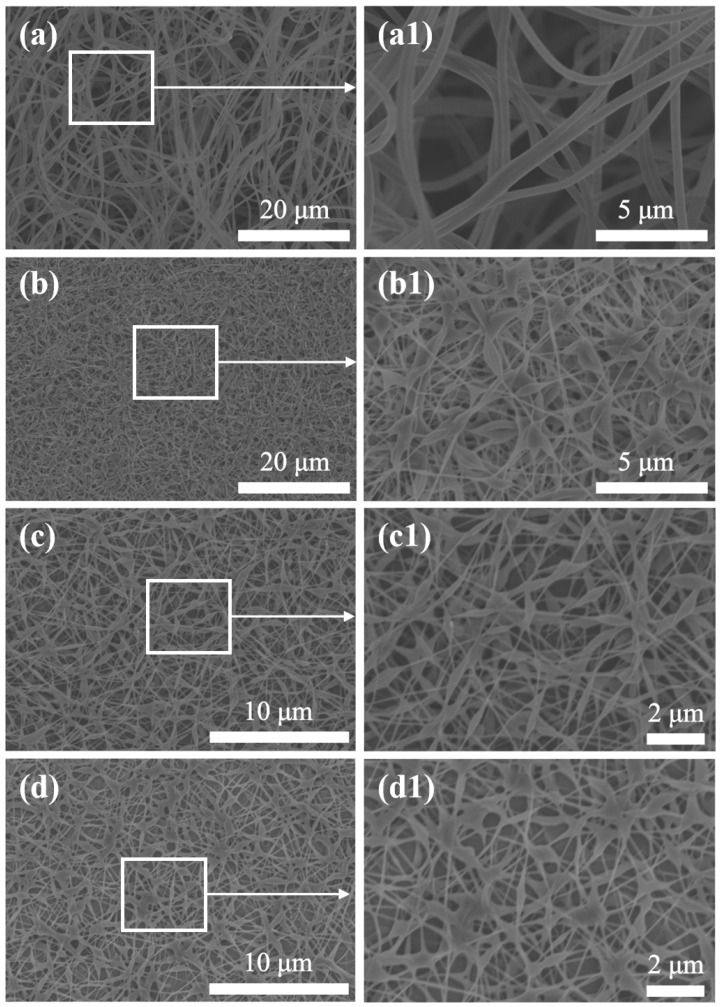
Scanning electron microscopy images of (**a**,**a1**) PVA, (**b**,**b1**) PVA/GA, (**c**,**c1**) PVA/GA/CNC, and (**d**,**d1**) modified PVA/GA/CNC composite membrane.

SEM was used to observe the microstructure of different PVA-based membranes. As shown in Figure 3a, pure PVA electrospun membrane exhibited a porous structure, which was suitable for the filtration of PM2.5. However, as shown in Figure 3(a1), the pore diameters of pure PVA electrospinning membrane were very uneven, and many pores were larger than 2.5 μm, which increased the passage rate of PM2.5 to reduce the filtration efficiency. To reduce pore diameter and obtain a denser porous structure, GA was introduced into the PVA-based membrane, which also enhances the filtration efficiency of PM2.5. As shown in Figure 3(b,b1), the internal pores of the PVA/GA membrane have a diameter of less than 1 μm. However, these small pores would cause an increase in the pressure drop of the filtration membrane and affect its application. As shown in Figure 3(c,c1), after the introduction of CNC into the PVA/GA mixture, the pore diameter of the resulting composite membrane can be increased to 2–3 μm. To make the composite membrane hydrophobic, a thermochemical deposition of MTMS vapor was performed. As shown in Figure 3(d,d1), there was no significant difference between the morphology of the modified composite membrane and that of the unmodified one, indicating that the deposition of a small amount of MTMS would not affect its morphology.

**Figure 4:**
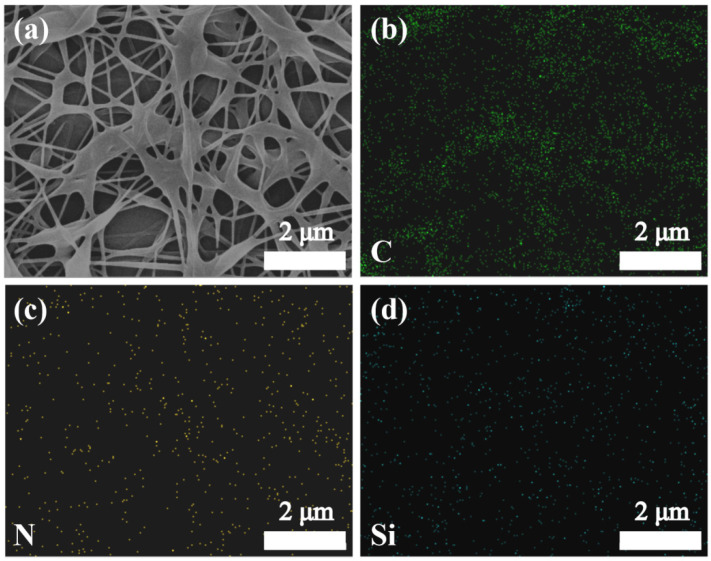
SEM image of (**a**) modified PVA/GA/CNC composite membrane corresponding to EDS spectrum of elements (**b**) C, (**c**) N, and (**d**) Si.

To show the deposition of MTMS, EDS analysis was performed on a modified PVA/GA/CNC composite membrane. As shown in Figure 4a–d, element N comes from GA, and GA and PVA are combined and evenly distributed in a modified PVA/GA/CNC composite membrane. Element Si comes from MTMS, and it can be observed that there is a uniform element Si on the surface of a modified PVA/GA/CNC composite membrane, which also indicates that MTMS is evenly deposited on the PVA/GA/CNC composite membrane.

### 3.3. Chemical Composition Analysis

**Figure 5:**
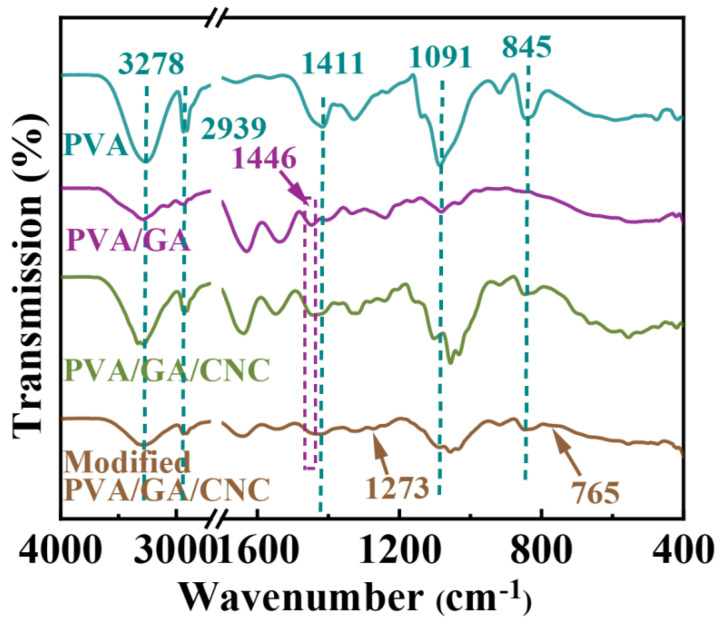
FTIR spectra of different PVA−based membranes.

The chemical structures of different PVA-based membranes were analyzed by FTIR (Figure 5). The pure PVA electrospinning membrane exhibited -OH stretching vibration at 3278 cm^−1^, -C-O bending vibration at 1091 cm^−1^, -CH_3_ bending vibration at 1411 cm^−1^, and -CH_2_ symmetric stretching vibrations at 2939 and 845 cm^−1^ [34,35]. After the introduction of GA into the system, the PVA/GA electrospinning membrane exhibited a C-N vibration peak at 1446 cm^−1^, which was caused by the vibration stretching of the C-N chain segment in GA [36,37,38]. After the introduction of CNC into the system, the PVA/GA/CNC composite membrane did not show a new vibration peak because no new chemical bond was introduced into the system [39]. After the thermochemical vapor deposition of PVA/GA/CNC composite membrane with MTMS, the system exhibited Si-C and Si-O-Si asymmetric stretching of MTMS at 1273 cm^−1^ and 765 cm^−1^, indicating the successful composite of MTMS [40,41]. Above all, it can be seen that the internal of the modified PVA/GA/CNC composite membrane mainly depends on hydrogen bonds or physical connections.

### 3.4. Thermal Stability Analysis

**Figure 6:**
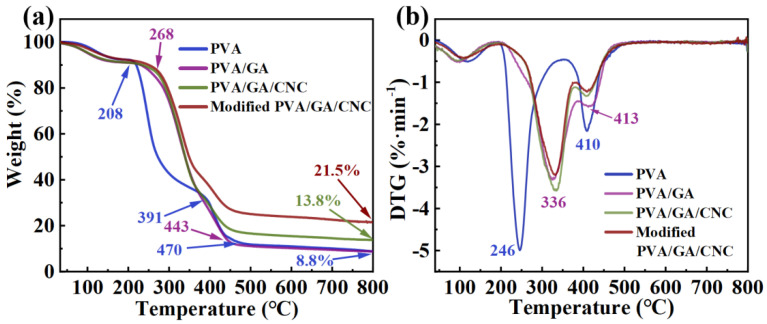
(**a**) TGA and (**b**) DTG curves of PVA−based composite membranes.

As shown in Figure 6a,b, all PVA-based electrospinning membranes had almost no mass loss before 100 °C, indicating that the absorbed water of the PVA−based electrospinning membrane evaporated during electrospinning. The mass loss of pure PVA electrospinning membrane between 100 and 208 °C was due to the removal of inside crystallized water. Then, the weight of the pure PVA electrospinning membrane decreased rapidly when the temperature was raised from 208 °C to 391 °C, and the fastest thermal decomposition rate was 249 °C, which was caused by the decomposition of the side chain of the PVA molecular chain. When the temperature was raised from 391 °C to 470 °C, the main chain of PVA was broken, and the weight decreased slowly, with a carbon residue of 8.8% at 800 °C [42]. After the introduced GA, the chain segment of GA decomposed rapidly with the chain segment of PVA in the process of 268 °C to 443 °C, and the added GA increased the thermal decomposition temperature of the system, indicating that GA had a significant effect on the thermal stability of PVA-based electrospinning membrane [43]. After the introduction of CNC into the system, the PVA/GA/CNC composite membrane showed a thermal decomposition curve similar to that of the PVA/GA electrospinning membrane, with a final carbon residue of 13.8% at 800 °C. The thermal decomposition curve of the modified PVA/GA/CNC composite membrane was similar to that of the unmodified one, and the final carbon residue at 800 °C was 21.5%. It was indicated that modified PVA/GA/CNC composite membranes have excellent thermal stability.

### 3.5. Mechanical Property

**Figure 7:**
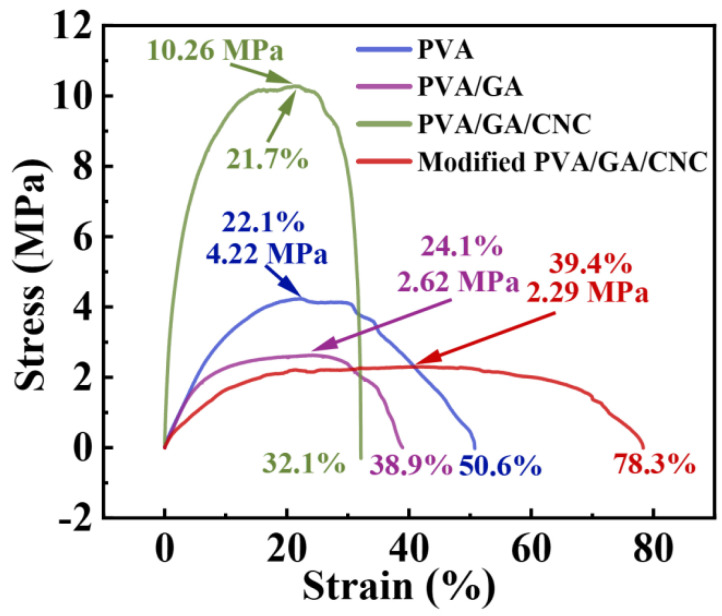
Tensile stress−strain curves of PVA, PVA/GA, PVA/GA/CNC, and modified PVA/GA/CNC composite membranes.

The mechanical properties of different PVA-based electrospinning membranes are one of the important standards for evaluating air filters. As shown in Figure 7, the pure PVA electrospinning membrane reached its maximum tensile stress of 4.22 MPa when stretched to 22.1% and broke at 50.6%. With the introduction of GA, the PVA/GA electrospinning membrane decreased its maximum tensile stress of 2.62 MPa when stretched to 24.1% and broke at 38.9%. Although GA can improve the thermal stability of the system, it has a certain effect on the mechanical properties of the system due to its greater brittleness. So, CNC was further introduced to enhance the maximum tensile rate and tensile stress of the system, and the PVA/GA/CNC composite membrane reached its maximum tensile stress of 10.26 MPa when stretched to 21.7%. However, this reduced the maximum elongation at break to 32.1%, affecting its practical useability. After hydrophobic modification with MTMS, the system reduced the maximum tensile stress, but enhanced the toughness, with the maximum stress reduced to 2.29 MPa and the maximum elongation at break increased to 78.3%. This may be due to the introduced MTMS, which is coated on the surface of the to enhance the toughness of the system.

### 3.6. Filtration Capacity

**Figure 8:**
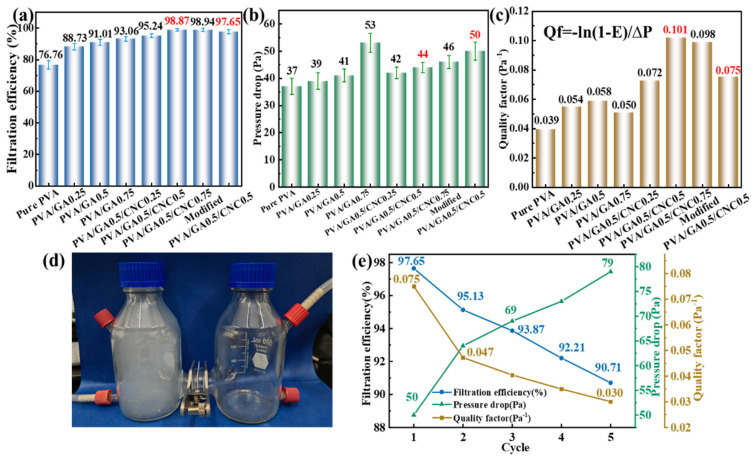
(**a**) Filtration efficiency, (**b**) pressure drop, and (**c**) corresponding quality factor of different PVA−based composite membranes for PM2.5. (**d**) Simple diagram of the PM2.5 filtration process. (**e**) The state of water droplets on the surface of modified PVA/GA/CNC composite membrane.

As shown in Figure 8a, the filtration efficiency for PM2.5 of pure PVA electrospinning membrane is 76.76%. This is because after electrospinning, the interior of PVA shows a spider-like network structure, which is limited by the diameter of three-dimensional pores, allowing part of PM2.5 to pass through the electrospinning membrane and reducing its capture rate of PM2.5. Also, due to its relatively loose pore structure, pure PVA electrospinning membrane shows a low-pressure drop (37 Pa). To improve the filtration efficiency of the system, GA was introduced to improve the density of the three-dimensional structure, and the results were consistent with those of SEM. With the introduction of GA, the filtration efficiency of the system is improved. In the PVA/GA electrospinning membrane, when PVA:GA = 1:0.25, the filtration efficiency for PM2.5 is 88.73%, and when the amount of GA is increased to PVA:GA = 1:0.75, the PM2.5 filtration efficiency is 93.06%. With the introduced GA, the pressure drop of the system increases. After the calculation of the quality factor (Qf = −ln(1 − E)/∆P), the optimal ratio of PVA/GA electrospinning membrane is PVA:GA = 1:0.5. However, even if the filtration efficiency for PM2.5 of PVA/GA electrospinning membrane reaches more than 90%, there is still room for further improvement. Therefore, CNC with a rich negative charge is introduced into the system, and the rich negative charge of CNC is used to attract PM2.5 with positive points. The results show that the introduced CNC has a great help in improving the filtration efficiency of the system, and the improvement effect of pressure drop is small. Because CNC is a short rod-like crystal, the introduced CNC in the system will not change its internal structure and improve the filtration efficiency for PM2.5 through electrostatic attraction. With the introduced CNC, PM2.5 filtration efficiency and pressure drop have been further improved, as shown in Figure 8c. According to the quality factors, PVA:GA:CNC = 1:0.5:0.5 is selected as the best ratio. In order to improve the wettability of PVA/GA/CNC composite membranes, a layer of hydrophobic MTMS was deposited on the surface of the films by vapor deposition. MTMS coverage reduced PM2.5 filtration efficiency (97.65%) and increased pressure drop (50 Pa), but the modified PVA/GA/CNC composite membrane still maintained the higher filtration efficiency and lower pressure drop. This also indicates that the modified PVA/GA/CNC composite membrane is an excellent PM2.5 filter material.

The PM2.5 filter device is shown in Figure 8d. Additionally, the hot gas deposition gives the system excellent hydrophobic ability, and the water contact angle reaches 101.9°. The modified PVA/GA/CNC composite membrane can remove surface adhering PM2.5 by washing to improve its filtration efficiency. As shown in Figure 8e, after five filtration–washing–re-filtration cycles, the modified PVA/GA/CNC composite membrane still has a high PM2.5 filtration efficiency (90.71%). The accumulation of PM2.5 in the modified PVA/GA/CNC composite membrane blocked the network structure in the membrane, resulting in an increased pressure drop (79 Pa). However, in general, modified PVA/GA/CNC composite membrane still maintains a high PM2.5 filtration effect after five washing cycles and could be used as an excellent air filtration device for personal protection, home or factory air purification, and other fields.

**Figure 9:**
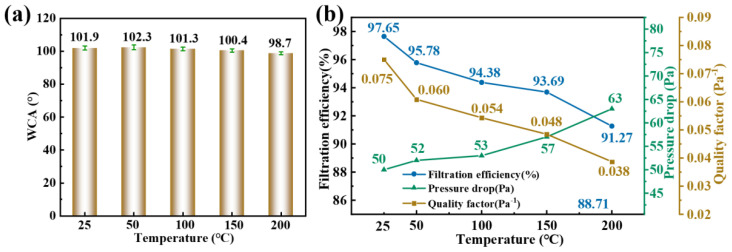
(**a**) WCA and (**b**) PM2.5 filtration performance of modified PVA/GA/CNC composite membrane at 25, 50, 100, and 150 °C, respectively.

The thermal stability of PM2.5 air filters is very key to their application in high-temperature scenarios, such as automobile exhaust filtration, high greenhouse, power plant chimneys, etc. After the recombination of GA in the system, the modified PVA/GA/CNC composite membrane showed good heat resistance in the TG. To show that modified PVA/GA/CNC composite membrane has strong heat resistance. In this study, the modified PVA/GA/CNC composite membrane was placed at 25, 50, 100, and 150 °C for 2 h and then removed to measure its WCA and PM2.5 filtration efficiency. As shown in Figure 9a, with the increased temperature, the WCA of the modified PVA/GA/CNC composite membrane gradually decreased but did not decrease significantly and still maintained a high hydrophobic capacity, which was due to the strong thermal stability provided by GA for the system. As shown in Figure 9b, with the increased temperature, the PM2.5 filtration performance of modified PVA/GA/CNC composite membrane gradually decreases (from 97.65% to 91.27%), which is the reason why PVA gradually melts with the increase in temperature. The pressure drop of the system also gradually increased (from 50 Pa to 63 Pa). However, the introduced GA alleviates this phenomenon so that the system still has a strong PM2.5 filtration capacity after high-temperature treatment. It is indicated that a modified PVA/GA/CNC composite membrane is an ideal PM2.5 filtration material for use in high-temperature environments.

## 4. Conclusions

In this work, PVA, GA, and CNC were mixed to prepare biomass-based membranes by electrospinning, and their surface wettability was modified by the thermochemical vapor deposition method. Among them, PVA was the main material, GA and CNC were the synergistic functional materials, and MTMS was the hydrophobic modifier. The modified PVA/GA/CNC composite membrane with a three-dimensional network structure was prepared. The system showed high toughness (maximum tensile fracture rate was 78.3%), hydrophobicity (101.9°), high filtration efficiency (97.65%), and low-pressure drop (50 Pa). It is an ideal material for air filtration due to its excellent air filtration performance. In addition, the modified PVA/GA/CNC composite membrane maintained a high level of air filtration performance (air filtration efficiency of 90.71% and pressure drop of 79 Pa) after five washing cycles. It maintains high air filtration performance at high temperatures (air filtration efficiency of 91.27% and pressure drop of 63 Pa in 200 °C). Given the environmental design philosophy of biomass-degradable materials, this study provides a simple and environmentally friendly strategy for constructing high-performance air filtration materials.

## Data Availability

The data presented in this study are available on request from the corresponding authors.
